# Effects of fishery complementary photovoltaic power plant on radiation, energy flux and driving forces under different synoptic conditions

**DOI:** 10.1038/s41598-023-36314-x

**Published:** 2023-06-05

**Authors:** Peidu Li, Xiaoqing Gao, Zhenchao Li, Tiange Ye, Xiyin Zhou

**Affiliations:** 1 Plateau Atmosphere and Environment Key Laboratory of Sichuan Province, School of Atmospheric Sciences, Chengdu University of Information Technology, Chengdu, 610225 China; 2 Key Laboratory of Land Surface Process and Climate Change in Cold and Arid Regions, Northwest Institute of Eco-Environment and Resources, Chinese Academy of Sciences, Lanzhou, 730000 China; 3 College of Resources and Environment, University of Chinese Academy of Sciences, Beijing, 100049 China

**Keywords:** Atmospheric science, Environmental impact

## Abstract

The underlying surface was the important media of air-lake interaction by transferring energy. The deployment of photovoltaic arrays on the lake has formed a new underlying surface type. But the new underlying surface is different from the natural lake. The impact of fishery complementary photovoltaic (FPV) power plants on the radiation, energy flux, and driving force is unclear. Therefore, the analysis of radiation, energy flux, and driving force by comparing the difference in the two sites under various synoptic conditions. The results indicated that the radiation components are not significantly different in the two sites under diverse synoptic conditions. The downward shortwave radiation (DSR) and net radiation (${R}_{n}$) were presented with one peak on a sunny day. The daily average DSR and Rn in the two sites were 279.1 W·m^−2^, 209.3 W·m^−2^, respectively. The daily average (cloudy day and rainy day) sensible heat flux in the two sites was 39.5 W·m^−2^ (FPV site), 19.2 W·m^−2^ (REF site), respectively. The latent heat flux was 53.2 W·m^−2^ and 75.2 W·m^−2^ on counterpart. The water body generally absorbs heat from the air (daily average ∆Q was 16.6 W·m^−2^) in the FPV site on a sunny day. The driving force of sensible heat flux in the FPV site was governed by the temperature of the FPV panel under sunny and cloudy conditions. The latent heat flux was determined by the product between wind speed and water-atmosphere temperature difference.

## Introduction

The utility-scale photovoltaic (PV) power plant is accelerating to achieve carbon peaking and carbon neutrality goals in China. The development of PV plants occupies a large amount of land resources that are important to the Chinese. Meanwhile, the land-use pattern, the underlying surface, and land–atmosphere energy transmission have been altered by the presence of utility-scale PV plant^1^. Therefore, the original balance of radiation and energy in the local region was broken by the deployment of utility-scale PV plant. Synoptic conditions immediate vicinity PV plant were affected by the change of original level of radiation and energy. However, few studies have been reported in these aspects. Studies of the impact of PV plant on local microclimate are accelerating in this disquisition direction. Taking temperature as an example, different scholars’ analyses of the impact of PV plant on temperature are shown in Table 1. The relationship of between meteorologic factors and PV plant in land were studied in these references. The total installed power generation of PV plant is accelerating in recent years. But the studies of the impact of PV plant in lake on radiation and energy were less reported. Meanwhile, the underlying surface of PV in land is significantly different from those in lake. The fishery complementary photovoltaic (FPV) power plant is a new type of using solar energy by PV power plant in China. The studies of the impact of FPV on the balance of both radiation and energy flux have been less presenting. In addition, the characteristic of radiation flux after installing FPV panels in lake under different synoptic conditions is unclear. Therefore, the mechanism of between the variation of radiation flux and its driving force after deployment of FPV panels in lake for disparate synoptic conditions were explored to promote the sustainable PV industry development.

**Table 1:** Research results of the impact of PV power plant on temperature.

Literature	Location	Time span	Conclusions
^2^	/	One year	Photovoltaic modules have a self-cooling function, which is particularly prominent at night
^3^	158° E–202° E	2010.08.14–2011.03.14	Under the self-cooling mechanism of the photovoltaic array, there is no heat island effect at an altitute of 5–18 m in the station during the day
^4^	The Los Angelse region	/	Considering the conversion efficiency of solar energy in the future, the deployment of solar photovoltaic can cool the urban environment
^5^	36°7′9″ N,100°32′22″ E	2015.08.01–2015.09.30	During the day, the temperature inside the station is higher than outside the station, and the adverseness is the case at night; the relative humidity of the air at 2 m at night changes significantly higher than outside the station
^6^	The Sahara region	/	Using a climate model with dynamic vegetation to simulate the coverage of wind farms and solar farms in the Sahara Desert will increase local temperature and precipitation by more than 2 times. This local enhancement depends on the scale of the electric field and is unique to the Sahara Desert. The impact in other deserts is small
^7^	36.136°N,100.588°E	2015.05–2016.04	Photovoltaic power plants are energy sinks throughout the year, especially in warm reasons
^8^	32°33′16.6″ N,111°17′03.7″ W	2017.09.26–2018.07.11	During the day, the daily average maximum temperature at 1.5 m inside the station was higher than that outside the station by 1.3 °C, and there was no significant difference in night temperature

/ represents no data information in the literature.

Eddy Covariance (EC) has few theoretical assumptions in the calculation process for the measurement of water and heat flux^9^, which provides the possibility for direct observation of the flux on the required time and space scale^10^. It is the most accurate method when the underlying surface is uniform and the terrain is flat and the atmospheric conditions are stable^11^. Therefore, this method has been widely used in actual observations. Sun et al.^12^ analyzed the energy flux and water evaporation changes of lakes in the Badain Jaran Desert through one year (2012.03–2013.03) EC observational data. The results showed that the daily and seasonal characteristics of long-wave and short-wave radiation fluxes change is obvious. The average annual evaporation rate of the lake is about 4.0 mm·d^−1^, the cumulative annual evaporation rate is 1445 mm·a^−1^, and the cumulative annual evaporation is 10 times the cumulative annual precipitation. Potes et al.^13^ analyzed the lake-atmosphere interaction in Alqueva reservoir in the summer of 2014. During the study, the energy of the reservoir was mainly released in the form of sensible heat flux and latent heat flux. Xiao et al.^14^ studied the control mechanism of the interannual variability of subtropical lake evaporation, answered the reasons for the change of lake evaporation under the scene of climate warming based on the EC measurement data and the principle of energy balance. It shows that lake evaporation increases with the amount of the solar radiation absorption and incident long-wave radiation. In addition, the decrease in lake evaporation is mainly caused by the feedback effect, in which weaken the reflected long-wave radiation. Spank et al.^15^ analyzed the EC observation data of the largest drinking water Rappbode Reservoir in Germany for one season. The results showed that the diurnal variation characteristics of the sensible heat of the reservoir are different from that of the land surface. At night and during the day, the latent heat flux and evaporation are abnormally low in the waters where evaporation is not restricted. There are a lot of studies on lake radiation characteristics and fluxes using EC observation data, which provides the possibility to further explore the lake energy flux response and lake-air interaction under the background of climate change. On the one hand, the change of energy flux was analyzed by the EC due to the efficient accuracy and widespread use. In addition, the few studies of energy flux for FPV power plant were less reported. Therefore, the characters of energy flux in FPV power plant were dissected by the EC data to reveal the impact of PV panels deployment on lake surface energy balance in this paper. FPV power plant is a new type of using solar energy by deployment of solar panels on water surface. The development of FPV power plant is a make a breakthrough at harnessing solar power field because of the installed region without the land limitation. However, there is a big difference of property between solar panels and lake underlying surface. That is an integrated underlying surface after installing the solar panels on original area. Solar radiation and energy balance in local area were affected by the deployment of FPV power plant. It is well-known that the lake-atmosphere interaction is dominated by the balance of both radiation and energy. The change of radiation and energy balance further conducted on local synoptic condition. At present, there are many studies on the variation of radiation flux and energy flux in PV power plants^7,8,16^. But the characteristic of radiation and energy balance under different synoptic conditions for FPV power plant was less reported. Hence, our study fills this research gap maybe to amend the accuracy of solar radiation prediction due to our paper to consider the impact of weather condition on solar radiation.

The characteristics of radiation and energy flux under different synoptic conditions for FPV power plant were analyzed in our paper. Meanwhile, the energy flux and its driving force and major environmental parameters were given to enhance understanding the interactive function between water surface and atmosphere after installing the PV arrays on lake. In addition, this study maybe helped to enhance the recognition of the impact of microscale climate on the solar radiation prediction.

## Materials and methods

### Study area and experimental equipment

**Figure 1:**
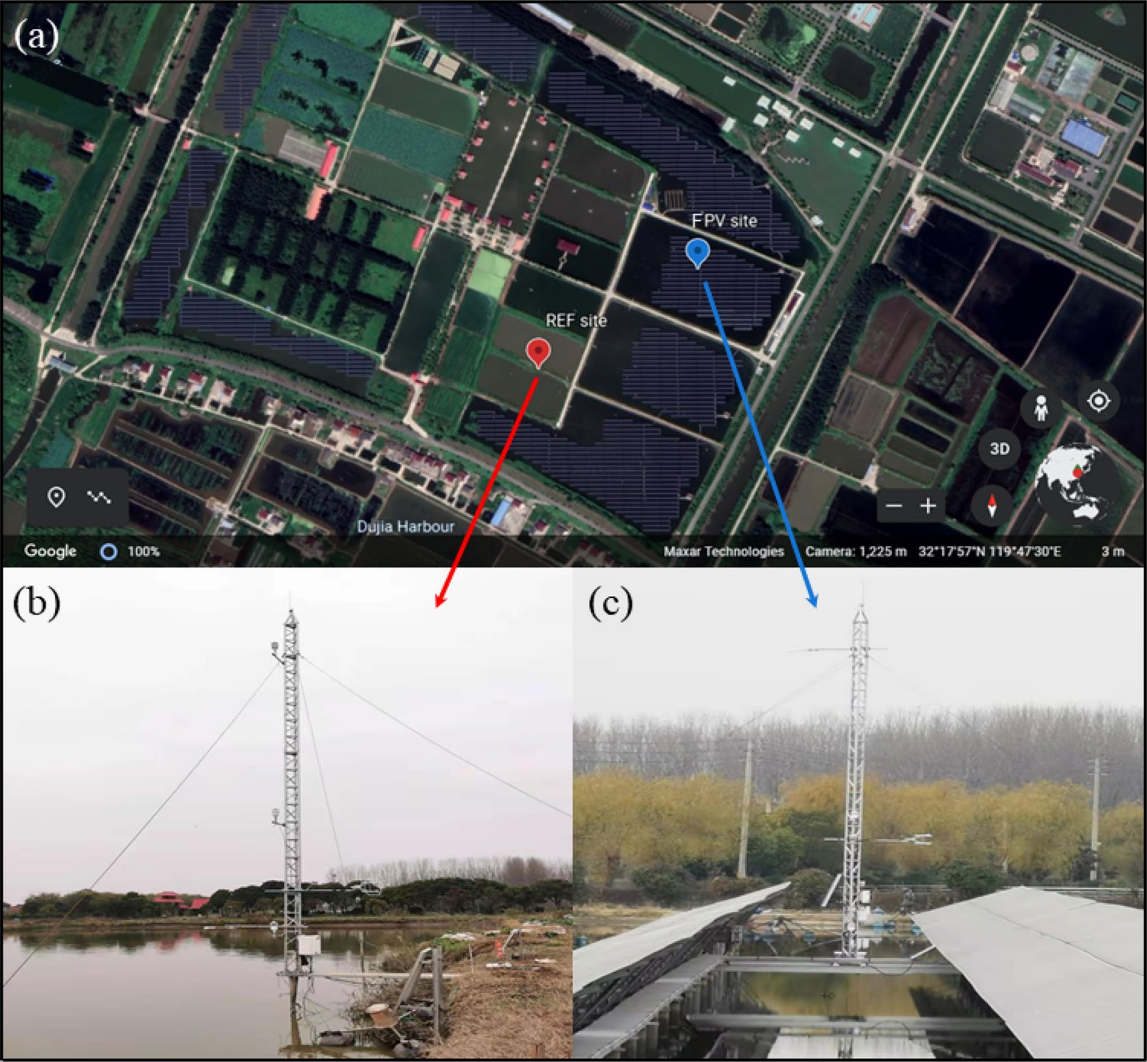
The study area location of the complementary photovoltaic power plant in Yangzhong, Jiangsu Province, China (**a**). The flux observational tower was marked by a pin, the blue pin was the FPV site, and the red pin was the REF site. The photo of the tower for the FPV site was shown in (**b**) and for the REF site was shown in (**c**).

The trial was conducted on the Tongwei Huantai 10 MW Fishery Complementary Photovoltaic Demonstration Base. This base is located on the Yangzhong City, Jiangsu Province of Eastern China. Yangzhong is situated in the middle of the northern subtropical monsoon climate zone, with a mild climate, abundant rainfall and the same season of rain and heat. From January to December in 2019, the average temperature for Yangzhong was 17.1 °C, the annual precipitation was 791.8 mm, and the annual accumulated sunshine time was 1792.2 h. The changes of radiation and energy flux installed PV arrays on the lake were captured by comparing the results from the flux observation tower inside and outside FPV power plant. The study area and the location of flux observation tower were shown in Fig. 1. The central coordinates of study area 32°17′5′′ N, 119°47′39′′ E, and the altitude is 2 m. The fishery complementary photovoltaic demonstration base is composed of four ponds of 5.7–8.9 acre. The FPV is located on the central the pond with about the water depth from 2.5 m to 3 m. The distance between the flux observational tower inside FPV power plant (the FPV site, blue pin) and outside flux observational tower (the reference site, abbreviate it to REF site, red pin) is about 251 m by Google Earth as shown in Fig. 1a. The details of two tower as shown in Fig. 1b (the FPV site) and Fig. 1c (the reference site).

**Figure 2:**
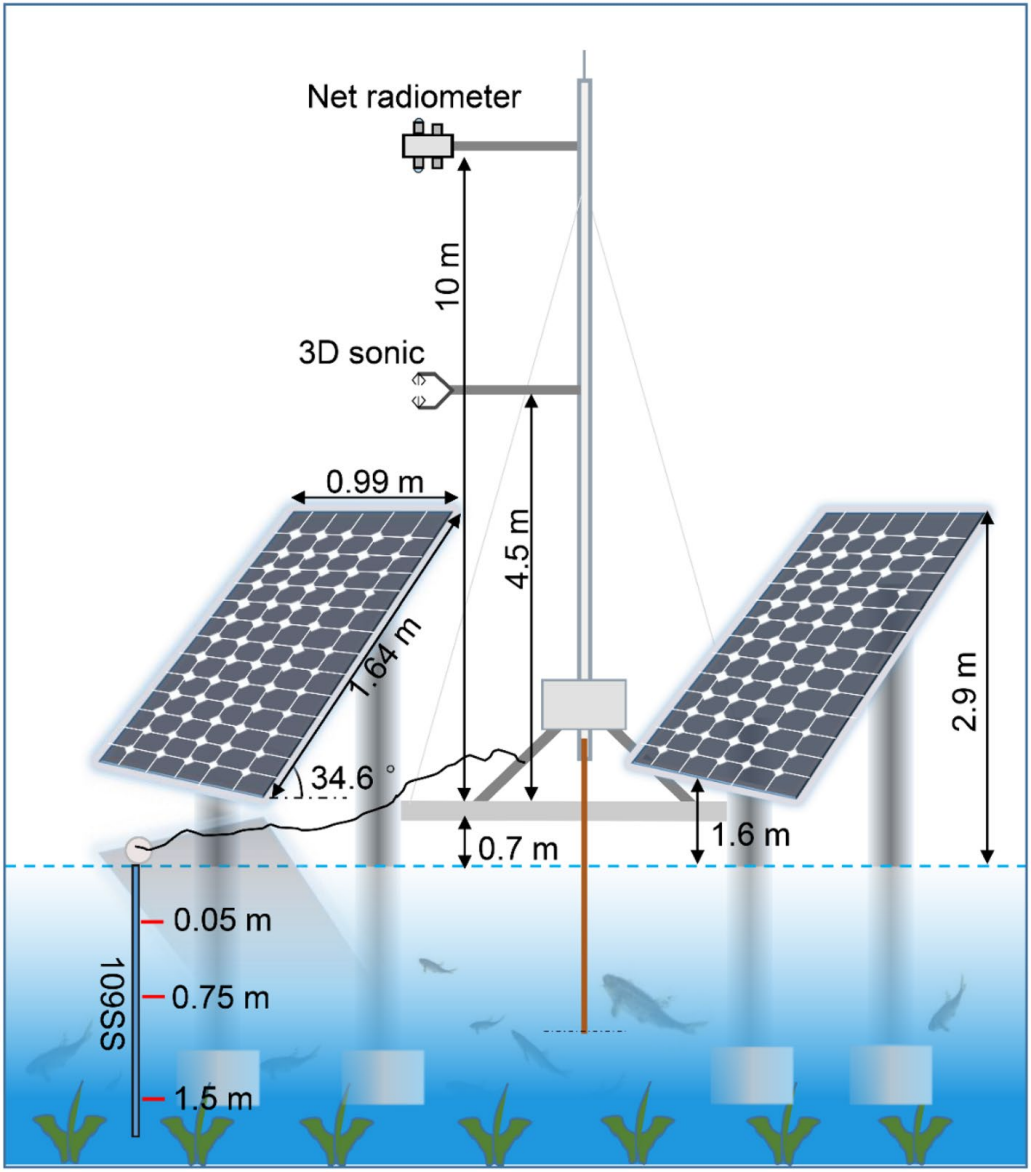
Schematic diagram of measuring devices in the FPV site.

The height of the flux observational tower is about 11 m for both two sites. The equipment of the flux observational tower for the FPV site is shown in Fig. 2. The equipment in FPV site is same to the REF site. The measuring devices included an Eddy-Covariance system (ECS), a net radiometer, three layers temperature sensor. But the installing height of the ECS and the net radiometer in the FPV site is higher than the REF site due to the PV panel's impact. The description details of measuring devices were recorded as follows. The FPV site coordination is 32°18′9.00′′ N, 119°47′33.45′′ E. The ECS (IRGASON-IC-BB, Campbell Scientific) was mounted on the tower for a height of 4.5 m. The installed height of the ECS for the FPV site is 2 m higher than the apex of the photovoltaic panel to avoid the horizontal wind uplift influence. The net radiometer (CNR4, Kipp & Zonen) was installed in the flux tower at a height of 10 m, the observation angle of the probe was 125°. The three water temperature sensors (109SS, Campbell Scientific) were placed depth at 0.05 m, 0.75 m, and 1.5 m, respectively. The water temperature sensors were tied to the buoy and rises or falls as the water level changes to ensure that the position of each probe from the water surface was basically unchanged. The REF site coordination is 32°18′4.60′′ N, 119°47′25.30′′ E. The measuring devices equipment in REF site is same as the FPV site. But the installing height of ECS and net radiometer was 3 m and 2 m without PV panels in the REF site, respectively.

Data collection of ECS starts on November 15, 2019, the high frequency data (10 Hz) are stored in a CR3000 data logger (Campbell Inc, USA) and half-hourly mean flux are calculated online by the Eddy Pro software. The measuring devices were maintained in time every month to ensure the accuracy and quality of data.

### Data processing

The half-hourly flux data was performed field removal, trend correction, secondary coordinate rotation, Gill ultrasonic anemometer angle correction, time delay correction, ultrasonic false temperature correction of sensible heat flux, frequency response correction, and water vapor correction by Eddy Pro software (v7.0.6). Finally, the average sensible heat flux (H) and latent heat flux (LE) were calculated according to the EC method, as follows formula (1) and (2).

1 $$H = \rho C_{p} \overline{{\omega^{\prime}\theta^{\prime}}}$$

2 $$LE = \lambda \rho \overline{{\omega^{\prime}q^{\prime}}}$$

where $\rho$(kg·m^−3^) is the air density, ${C}_{p}$ is the specific heat at constant pressure as 1004.67 J·kg^−1^·K^−1^. $\lambda$ is the latent heat of water (2.5 × 10^6^ J·kg^−1^). And the parameters ${\theta }^{^{\prime}}$(K),${q}^{^{\prime}}$(kg·kg^-1^) and ${\omega }^{^{\prime}}$(m·s^−1^) are the deviations from the temporal averages of the air temperature, the specific humidity and vertical wind speed, respectively.

As net radiometer is less affected by the weather, the raw data of downward shortwave radiation ($DSR$), upward shortwave radiation ($USR$), downward long-wave radiation ($DLR$) and upward long-wave radiation ($ULR$) need not be processed. Net radiation (${R}_{n}$) is calculated by the formula (3).

3 $$R_{n} = \left( {DSR - USR} \right) + \left( {DLR - ULR} \right)$$

A whole lake is a research object to evaluate the energy budget, the energy balance of a lake Eq. (4) can be expressed by the following formula^17^:

4 $$R_{n} - \Delta Q = H + LE + \Delta Q_{B} + \Delta Q_{F} + \Delta Q_{P}$$

where $R_{n}$ is the net radiation, $\Delta Q$ is the heat storage of lake water, $H$ is the sensible heat flux, $LE$ is the latent heat flux, $\Delta {Q}_{B}$ is the heat flux of lake sediments, $\Delta {Q}_{F}$ is the change in heat storage from runoff and through the lake, $\Delta {Q}_{P}$ is the change in heat storage caused by precipitation. The units of radiation and flux in our paper are W·m^−2^.

Because the lake is enclosed, there is no heat exchange caused by the exchange of inflow and outflow water bodies, so $\Delta {Q}_{F}$ can be ignored. The daily variation of $\Delta {Q}_{B}$ and $\Delta {Q}_{P}$ compared with $H$ and $LE$ can also be ignored, so the energy balance equation can be simplified to the following formula (5):

5 $$R_{n} - \Delta Q \approx H + LE$$

where ${R}_{n}-\Delta Q$ is the available energy, $H+LE$ is the turbulent energy flux.

The heat storage $\Delta Q$ of the water body is calculated based on the time change of the average temperature of the lake, as shown in the following formula (6):

6 $$\Delta Q = \rho_{w} c_{pw} \int\limits_{0}^{z} {\frac{{d\overline{T}_{w} }}{dt}z}$$

where ${\rho }_{w}$ is the density of the water (kg·m^−3^), ${c}_{pw}$ is the specific heat capacity of the water (4192 J·kg^−1^·K^−1^)^17^; $z$ is the maximum depth of measured water temperature profile on the lake (m).

The vertical profile of the water temperature change was obtained by the three layers (0.05 m, 0.75 m and 1.5 m) water temperature observation, then the depth-weighted average water temperature is calculated to obtain the depth-weighted average water temperature change within a fixed time step (30 min). The formula (7) is as follows:

7 $$\Delta \overline{T}_{w} = \frac{1}{z}\sum\nolimits_{i = 1}^{n} {\Delta T_{w,i} \Delta z_{i} }$$

where ${T}_{w,i}$ represents the average water temperature of the first $i$ layer, $\Delta {z}_{i}$ is the thickness of the water layer of the first $i$ layer.

## Results and discussion

### Radiation

**Figure 3:**
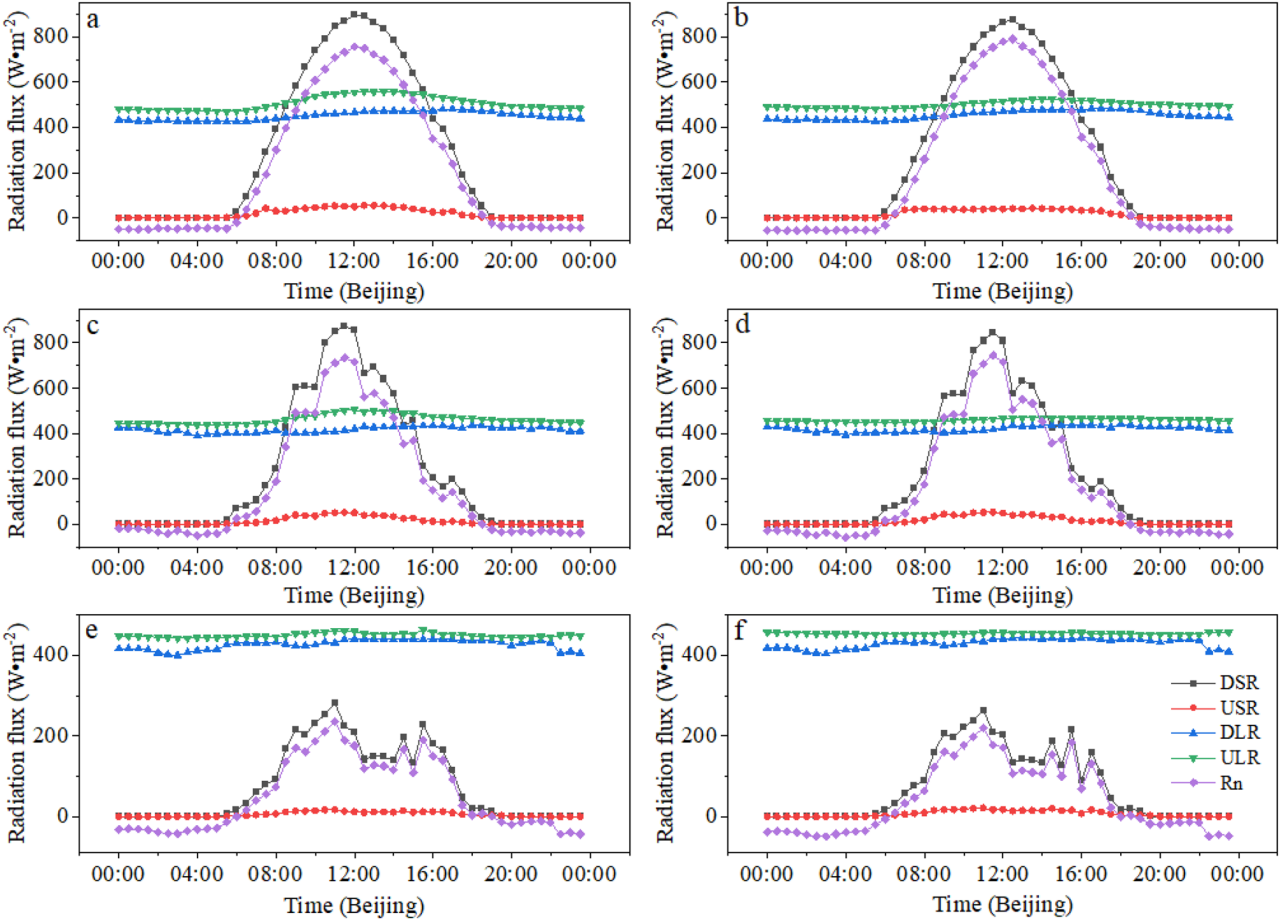
Daily radiation flux in the two sites (The first column is the FPV site, the second column is the REF site) under different synoptic conditions (Different rows stand for the various synoptic conditions, the first row is the sunny, the second row is the cloudy, the third row is the rainy).

The changes in daily radiant flux on the lake in two sites are shown in Fig. 3. In general, the diurnal variation of DSR, Rn, and USR is generally presented as an obvious peak type, with peaks appearing between 11:00 and 12:30 in Beijing. DLR and ULR are relatively more stable than other radiation components. And the changes of DSR and Rn are more synchronized, because the fluctuation of DLR, ULR, and USR was very small on the whole day.

August 16, 2020 was a typical sunny day, and the radiation flux curves in two sites were relatively smooth (Fig. 2a,b). Starting from 6:00, DSR gradually increases with solar radiation. The DSR peaks were 897 W·m^−2^ in the FPV site and 875 W·m^−2^ in the REF site at 12:30 and 12:00 in Beijing, respectively. DSR is mainly affected by meteorological elements such as cloud cover and aerosol^18,19^. The daily variation of USR for the FPV site is greater than the REF site because the albedo (albedo = USR/DSR) was increased (the FPV site albedo was 6.56%, the REF site albedo was 6.63%) on sunny day. But Li et al. found that the albedo is decreased after deploying the PV arrays on lake^20^. Because the albedo of lake is higher than the PV panel materials. Therefore, the water environmental quality of lake maybe improved by the deployment of PV arrays. The albedo is higher to cause the eutrophication of water bodies^21^. Additionally, the deployment of PV arrays on lake can also the change of the thermal properties in two sites, which is mainly presented in the difference from the long wave reflection. The daily minimum of ULR in FPV site was 470.4 W·m^−2^ at 5:30, and ULR minimum appeared 30 min later in REF site (482 W·m^−2^) than in the FPV site later 30 min.

With the solar radiation increasing, the ULR in the FPV site was greater than in the REF site at 7:30. The ULR daily peak in the FPV site appears at 12:30 as 559.2 W·m^−2^. The ULR daily peak in the REF site appears at 14:30 as 525.7 W·m^−2^. The diurnal range of long-wave radiation on the lake surface in the FPV site and in the REF site are 88.8 W·m^−2^ and 43.7 W·m^−2^, respectively. It can be clearly seen that the diurnal range of long-wave radiation in the FPV site was greater than that the REF site. For the net radiation, the difference in two sites was not obvious. The net radiation in the FPV site peaks at 12:00 as 757 W·m^−2^, and the net radiation in the REF site peaks at 12:30 as 790 W·m^−2^. The hour of the net radiation status that was positive (6:00) or negative (19:00) was the same in two sites, and the DSR change was also synchronized with the net radiation.

The variation of the radiation component was analyzed in two sites (Fig. 3c,d) for the cloudy day on July 1, 2020. The trend of DSR in two sites was synchronous change. DSR has appeared jagged characteristic in two sites from 8:30 to 15:00 due to the clouds influence. The average DSR in the FPV site and REF site was 226.48 W·m^−2^ and 214.10 W·m^−2^, respectively. The average USR of the FPV site was 1.4 W·m^−2^ smaller than that of the REF site. The DLR trends in two sites are synchronized well, and the average in FPV site and the REF site is 420.08 W·m^−2^ and 416.64 W·m^−2^, respectively. The maximum impact of PV arrays on the radiation component is mainly presented in the difference of ULR in two sites. The PV panel heats up rapidly than the water with the increase of solar radiation because the specific heat of the PV panel (950 J·kg^−1^·K^−1^)^22^ is smaller than that of the water (4184 J·kg^−1^·K^−1^). ULR in the FPV site had been reaching the peak of the day at 12:00 with 505.7 W·m^−2^. On the contrary, the peak in the FPV site was lagged than the REF site and it appears at 14:00 as 469.5 W·m^−2^. The variation of URL in FPV site was greater than that of the FPV site with the solar radiation was ebbing away. ULR in the FPV site decreases significantly after 18:00 with reaching the minimum at 4:00 a day as 437.6 W·m^−2^.

The rainy day was selected on July 6, 2020. The change curve of radiation components in the two sites is shown in Fig. 3e (the FPV site) and Fig. 3f (the REF site). DSR began to rise at 5:30 and reached the daily peak at 11:00 with 282.7 W·m^−2^ in the FPV site and 262.9 W·m^−2^ in the REF site, respectively. The daily variation of DSR presented a jagged fluctuation after the rain was starting at 11:00. In order to clearly compare the difference in radiation between the FPV site and the REF site, the variation of radiation components under different synoptic conditions was shown in Table 2. In general, the net radiation in the two sites decreased in the order of “sunny-cloudy-rainfall”, and other radiation components also present the same characteristics.

**Table 2:** Daily average radiant flux in the two sites under different synoptic conditions (W·m^−2^).

	The FPV site					The REF site				
	DSR	USR	DLR	ULR	Rn	DSR	USR	DLR	ULR	Rn
Sunny	272.9	17.9	454.4	500.4	209.0	285.2	18.9	450.1	506.8	209.5
Cloudy	214.1	15.6	420.1	459.4	159.2	226.5	14.2	416.6	462.9	166.1
Rainy	73.1	7.0	429.5	454.1	41.4	78.6	5.5	426.8	449.4	50.4

In general, the deployment of PV arrays on the lake is forming a new underlying surface. The radiation characteristic change of FPV (one peak) is the same as the desert^8,23^, grassland^24^, et al. The difference in radiation on various underlying surfaces is from the impact of aerosol, water vapor, terrain, and weather conditions on sunlight.

### Energy flux

**Figure 4:**
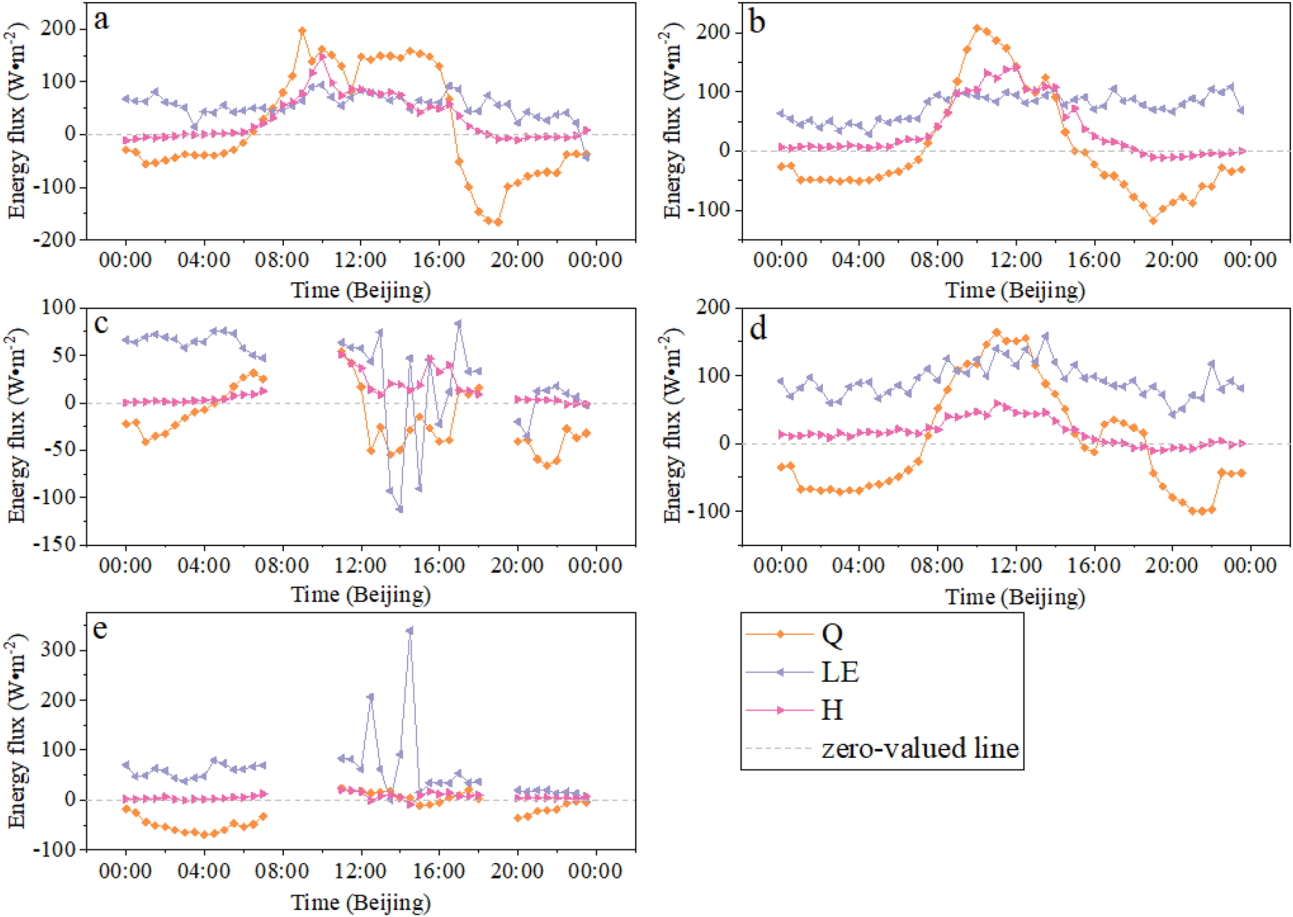
Daily variations of energy flux in the two sites (**a**–**c** stand for the FPV site; **d**,**e** stand for the REF site) under different synoptic conditions (**a**, sunny day; **b**, **d**, cloudy day; **c**, **e** rainy day).

The variation of energy flux changes in the two sites is shown in Fig. 4. Overall, the latent heat flux (LE) in the two sites was relatively stable, and the sensible heat flux (H) in the FPV site has more obvious fluctuations than that of the REF site when there is solar radiation. This phenomenon was explained by the air temperature in the FPV site rising faster due to the PV panel warming effect with obtaining the solar than the air temperature in the REF site. ∆Q has showed zigzag-like fluctuation. The positive or negative ∆Q means to absorb or release heat from the air to water. The value of ∆Q gradually becomes positive with increasing net radiation, which indicated that the water absorbs heat from the air during the PV panels working period. Instead, ∆Q is negative during the PV panels not working period which means the water releases heat to the air. The pattern of absorbing during the PV working period and releasing heat during the PV not working period after installing PV arrays on the lake has achieved the heat balance between water and air. The selection of a sunny day was very difficult because the study area was entering the rainy season after June 2020. On August 16, it was a sunny day and there was no cloud in the sky according to our meteorological data. It was pity that the energy flux data in the REF site was missing. Therefore, the energy flux in the FPV site was only analyzed here. The average of H and LE in the FPV site was 29.53 W·m^−2^ and 56.38 W·m^−2^, respectively. The H with an average value of only 0.3 W·m^−2^ was smaller than LE during weak solar radiation before 7:30. Then, the H was greater than the LE and this process lasted until 14:30 because the PV panels heats up continuously with the solar radiation. This duration was reflected by the influence of the DSR change process on the H, and the rate of the H change was determined by the properties of the photovoltaic panel^25^. The daily average of ∆Q was 16.6 W·m^−2^, which means the water body generally absorbs heat from the air. Especially ∆Q > 0 from 6:30 to 16:30 was the water storage heat phase and other timespans were the water release of heat phase. Therefore, this heat change process plays an important role in maintaining the stability of the water environment.

In cloudy weather, the average LE in the FPV site was 74.76 W·m^−2^, and the average LE in the REF site was 93.42 W·m^−2^. The LE in the FPV site was smaller than that of the REF site because the area on the lake of directing the solar radiation was reduced by the shading effect of the PV arrays. The overall change trend of the H in the FPV site was significantly greater than that of the REF site. But the solar radiation before 6:30 and after 19:00 was absent. Therefore, the H in the FPV site was smaller than that of the REF site. The H in the FPV site after 6:30increased with the solar radiation. it reached the daily maximum at 12:00 noon with 140.9 W·m^−2^. Subsequently the H decreased with the solar radiation decline. The change of H in the FPV site was significantly greater than that of the FPV site with the presence of solar radiation. The average of H in the FPV site and the REF site was 66.09 W·m^−2^ and 26.19 W·m^−2^ from 6:30 to 18:30, respectively. With the absence of solar radiation, the average of H in the FPV site and the REF site was 1.33 W·m^−2^ and 5.98 W·m^−2^, respectively. In general, the contribution of the impact of solar radiation on the H in the two sites was 98% (FPV site) and 77% (REF site), respectively. The impact of the PV array installation in the lake on the H is 1.5 times compared to the H of the natural lake. Therefore, solar radiation plays a decisive role in the change process of H for the FPV power plant.

The H data during the rainy weather period has been eliminated, and the average H was significantly lower than other synoptic conditions. The H of the FPV site and the REF site in the effective datum was 11.6 W·m^−2^ and 6.2 W·m^−2^, respectively. The variation of LE was the opposite of the rule of H. The LE in the two sites was 31.7 W·m^−2^ (FPV site) and 56.9 W·m^−2^ (REF site), respectively. ∆Q was negatively explained by the air temperature drop sharply for precipitation, which indicates that the air absorbs heat from the water body to achieve a water–air heat balance.

### Driving forces of energy flux in the two sites

**Figure 5:**
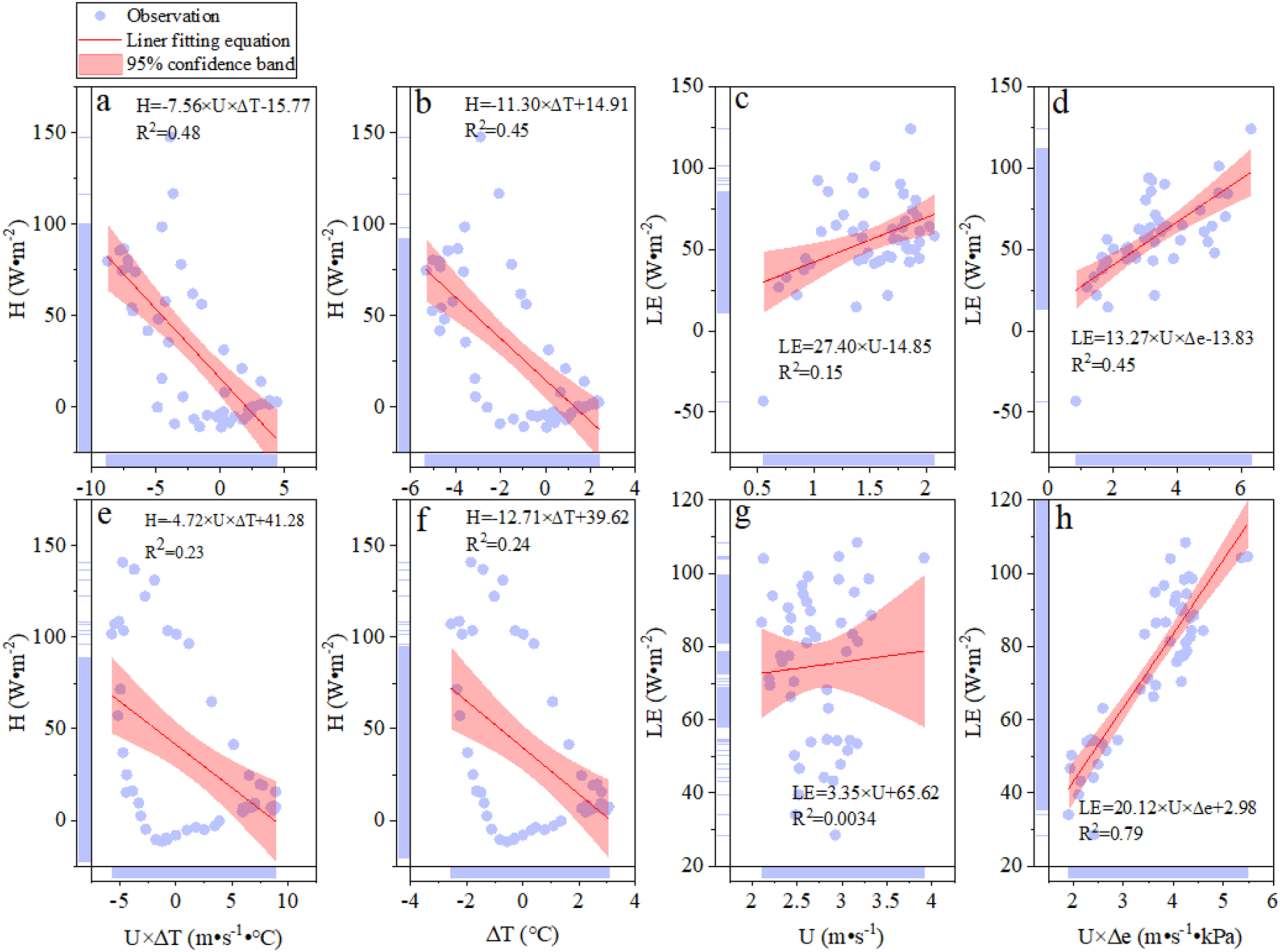
Driving force of energy flux in the FPV site under different synoptic conditions. The row information is stand for the different synoptic condition (the first row is the sunny, the second row is the cloudy). The column information is stand for the driving force. From left to right: U × ∆T, ∆T, U, and U × ∆e.

The relationship between the H, LE, and environmental factors in the FPV site under different synoptic conditions was shown in Fig. 5. The abbreviation of a proper noun and its unit in this part was given to improve the readability of the paper. Those abbreviations as follows: wind speed (U, m·s^−1^), water-atmosphere temperature difference (∆T, ℃), and water–air vapor pressure deficit (∆e, kPa). The correlation between H and some environmental factors (U × ∆T, and ∆T) was negative in the FPV site under sunny and cloudy conditions (Fig. 5a,b,e,f). The correlation between H and the U × ∆T (0.48) & ∆T (0.45) on a sunny day was twice as high as those on a cloudy (0.23 & 0.24). The result indicated that the driving force of the environmental factors U × ∆T and ∆T on a sunny day was greater than that of on a cloudy day for H. The LE was positive with some environmental factors (U & U × ∆e) on a sunny day and cloudy day (Fig. 5c,d,g,h) for the FPV site. The R^2^ between LE and U was 0.15 on a sunny day. But this relationship (R^2^ = 0.003) was very weak on a cloudy day indicating that the LE was not driven by the U. In addition, the correlation between LE and the U × ∆e was higher than that of the U under the same synoptic conditions. Therefore, the driving force of LE is dominated by the U × ∆e. In particular, the explanation of U × ∆e to the LE is close 80% under cloudy conditions.

**Figure 6:**
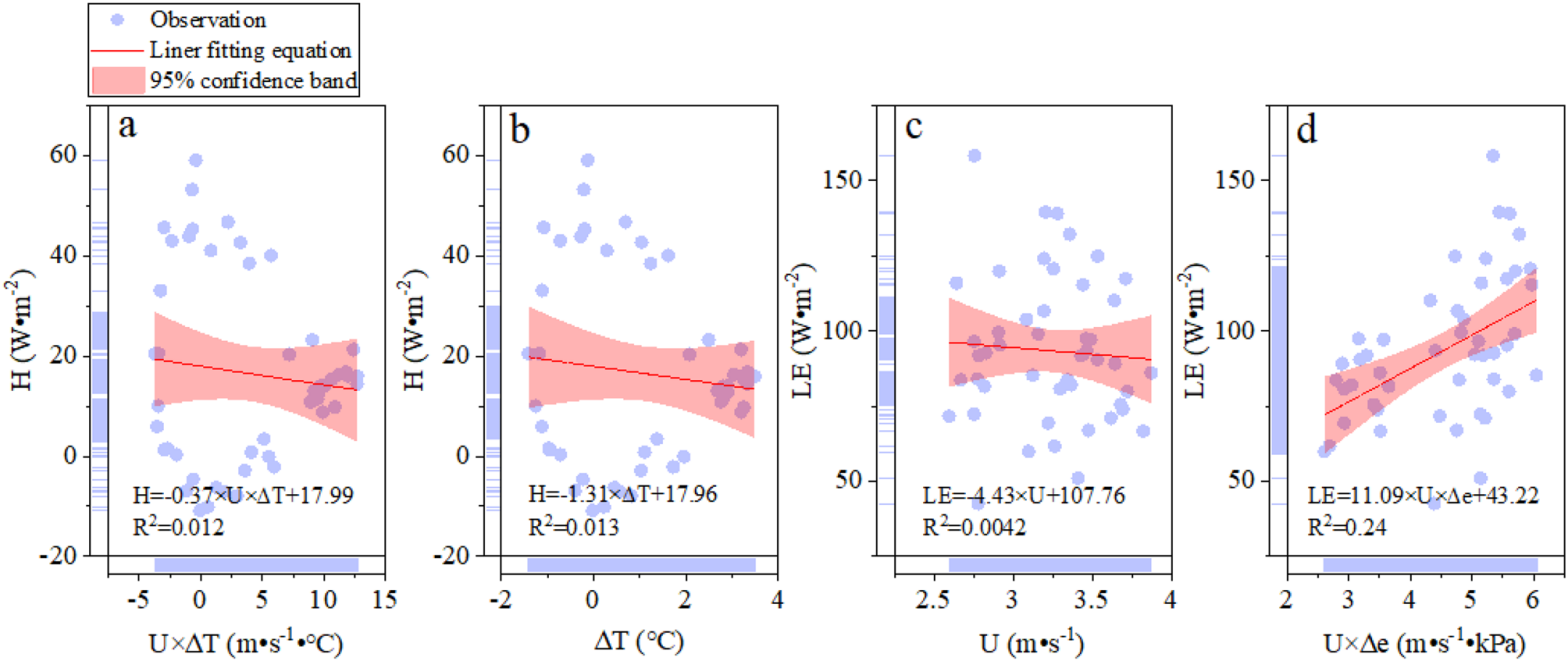
Driving force of energy flux ((**a**) U × ∆T; (**b**) ∆T; (**c**) U; (**d**) U × ∆e) in the REF site in the REF site on cloudy day.

The relationship between the energy flux (H & LE) and its driving force in the REF site on a cloudy day was shown in Fig. 6. The correlation of H and some driving force (U × ∆T and ∆T) was negative. In particular, the relationship of H and driving force (U × ∆T) was very weak (R^2^ = 0.012). The driving force of U × ∆T can only explain about 1% of the change in H. But the explanation of driving force U × ∆T was 23% (R^2^ = 0.23) for H in the FPV site under cloudy conditions. Because the shadowing effect of PV arrays affects the wind speed and the warming effect of arrays induces the air temperature. Therefore, the explanation of driving force U × ∆T was significantly improved for H after the deployment of PV arrays on the lake under the same weather conditions. The correlation between LE and U was negative, which was opposite to the result in the FPV site under the same weather conditions. In addition, the correlation between LE and U × ∆e was positive in two site under a cloudy condition. But the correlation of LE and U was different in the two sites. Therefore, the change of LE was not determined by the U and the multiple driving forces (U × ∆e) were functionary for LE. The H was driven by the U × ∆T and ∆T in the FPV site under sunny and cloudy conditions. And this correlation was weak in the REF site under cloudy conditions. Those results were contrary to the Nordbo et al.^17^ study that the H on a natural lake can be explained by the U × ∆T. The explanation of U × ∆T for the H was improved after deploying the PV arrays on the natural lake. The correlation between H and U × ∆T from the FPV site to the REF site was decreased gradually. Meanwhile, the correlation between LE and U × ∆e from the FPV site to the REF site presented the same change of H and some driving force, such as U × ∆T. The change in correlation between energy flux and driving forces indicated the impact of PV arrays on the local environment. The major driving force of dominating the LE was U × ∆e in the two sites under sunny and cloudy conditions. This result is consistent with the study of Du et al.^26^ on Erhai Lake in China, and this relationship is also found in lakes in Germany and France^17,27^. Because the PV arrays block off the airflow which leads to the reduction of the resistance of air. Meanwhile, the ∆e is increased with the airflow decrease. Therefore, the explanation of U × ∆e for LE in the FPV site was greater than that of the REF site.

In general, in Fig. 5 R^2^ is from 0.15 to 0.45 and in Fig. 6 R^2^ is 0.012 to 0.24. The correlation value is unsufficient. These meteorological factors for the driving force of energy flux were selected according to the Nordbo et al. results on natural lake^17^. The R^2^ is lower due to the underlying surface of the lake being changed by installing solar panels. Of course, our results indicated that the driving force of energy flux is affected by the deployment of solar panels on natural lakes. Therefore, the relationship between the driving force of energy flux and meteorological factors are explored by the correlation coefficient matrix to illustrate the effect of the deployment of solar panels on natural lakes on energy exchange.

**Figure 7:**
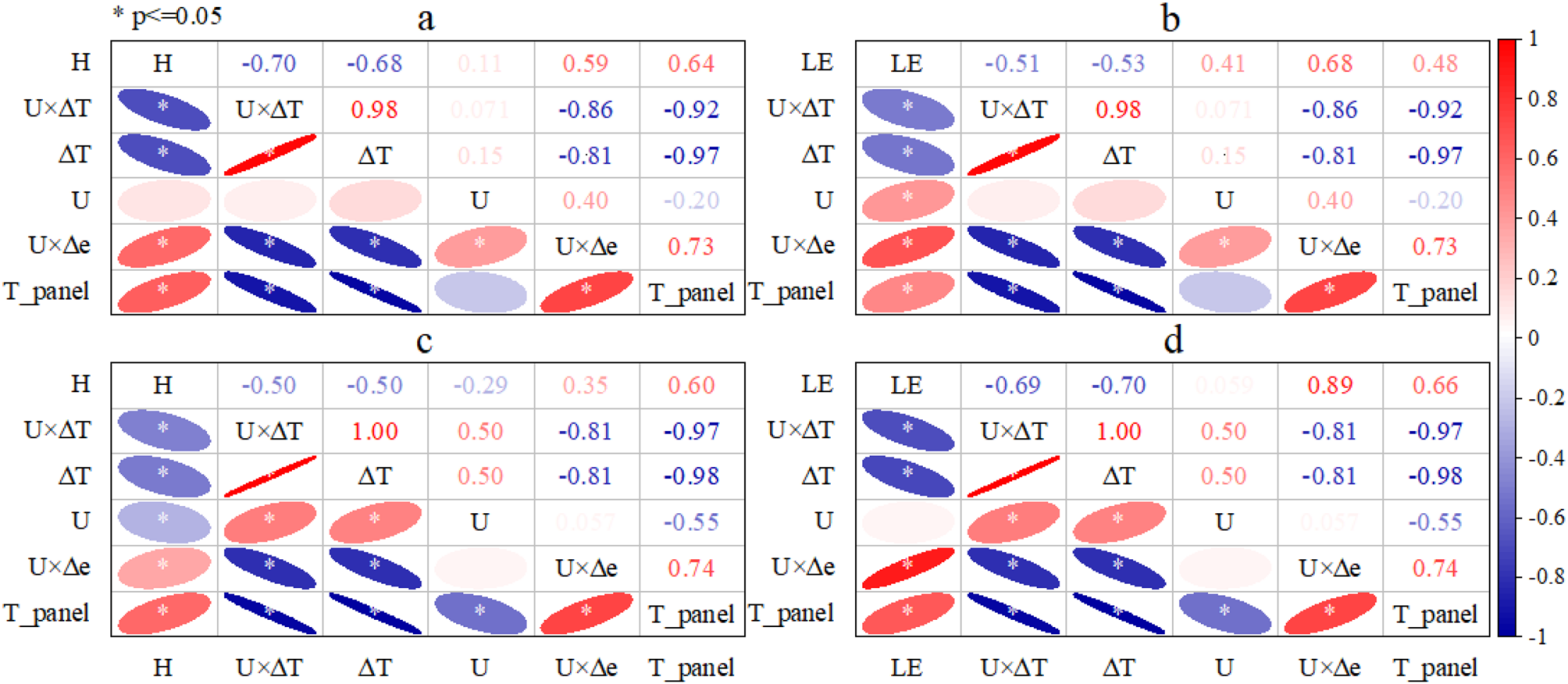
Pearson’s correlation coefficients in the FPV site between energy flux and environmental factors under different synoptic conditions. (**a**) Pearson’s correlation coefficients between H and environmental factors (U × ∆T, ∆T, U, U × ∆e, T_panel is PV temperature) on sunny day, (**b**) Pearson’s correlation coefficients between LE and environmental factors on sunny day, (**c**) Same as the (**a**), but the synoptic condition is cloudy, (**d**) Same as the (**b**), but the synoptic condition is cloudy. Red ellipse represents positive correlation, blue ellipse represents negative correction, and white asterisk represents the significance level of 0.05.

The driving force of energy flux in the FPV site is different from the natural lake. The correlation coefficient matrix between energy flux and environmental factors in the FPV site under different synoptic conditions is shown in Fig. 7 to seek out the major factor of energy flux. The H in the FPV site on cloudy conditions was firstly dominated by the PV temperature (T_panel). The correlation coefficient between H and T_panel was 0.64 and it was very significant. The second influencing factor of H was the U × ∆e (0.59). This factor is so much important because it was a robust relation (0.73) with the major factor of T_panel. On a cloudy day, the correlation coefficient between H and T_panel was slightly weaker than that on a sunny day (Fig. 7c, 0.60). But the correlation coefficient between H and T_panel is the strongest than others. Therefore, the T_panel is the substantial dominant influence factor of the H for the FPV site. The main controlling factor of LE is U × ∆e on sunny and cloudy condition for the FPV site. But the correlation coefficient between LE and U × ∆e on a cloudy day (0.89) was greater than that on a sunny day (0.68).

## Conclusions

The deployment of PV on the lake is forming a new type of underlying surface. The analysis of radiation, energy flux, and its driving force in the two sites to understand the interaction of air-lake boundary layer. In addition, the study of the difference in radiation under various synoptic conditions may improve the accuracy of solar forecasts on a short-term scale. The study analyzed the difference in radiation, energy flux, and driving force under different synoptic conditions by comparing the FPV site and the REF site. The following conclusions are derived from the situ observations and investigation:

1. The radiation components were significantly different in the two sites under various synoptic conditions. The DSR and Rn were presented with one peak on a sunny day. But the smooth characteristic curve in DSR and Rn is interrupted on other synoptic conditions. The daily average DSR and Rn in the two sites were 279.1 W·m^−2^, 209.3 W·m^−2^, respectively. In addition, the average ULR in the FPV site is 1.73 W·m^−2^ higher than the reference site.
2. The H change was fluctuant in the two sites under various synoptic conditions due to the impact of DSR on T_panel. The LE was relatively stable in the two sites under different synoptic conditions. The daily average (cloudy day and rainy day) H in the two sites was 39.5 W·m^−2^ (FPV site), 19.2 W·m^−2^ (REF site), respectively. The LE was 53.2 W·m^−2^ and 75.2 W·m^−2^ on counterpart. The water body generally absorbs heat from the air (daily average ∆Q was 16.6 W·m^−2^) in the FPV site on a sunny day.
3. The driving force of H in the FPV site is governed by the T_panel under sunny and cloudy conditions. The main driving force of LE is U × ∆e on sunny and cloudy condition for the FPV site.

The analysis of radiation, energy flux, and its driving forces under different synoptic conditions by comparing the two sites. But there are limits in this paper. The results were obtained by the selection of three days due to the observational data limitation. The data details in the study are shown in Supplementary file. The accuracy of results may be improved by the classification of observational data according to the weather conditions. In addition, the impact of the FPV power plant on the radiation and energy flux was related to its scale. The effects of utility-scale FPV power plants on the radiation and energy flux need to be further researched.

## Supplementary Information

Supplementary Information.

## Data Availability

All data generated or analyzed during this study are included in this published article and its supplementary information files.
